# Spontaneous Coronary Artery Dissection: A Challenging Diagnosis

**DOI:** 10.7759/cureus.47603

**Published:** 2023-10-24

**Authors:** Sayna Poursdarolah, Mariam Seliman, Jonathan Abaya Ghazaleh, Selvana Poursadrolah, Andrew Rubin

**Affiliations:** 1 Internal Medicine, Eisenhower Medical Center, Rancho Mirage, USA; 2 College of Medicine, Guilan University of Medical Sciences, Rasht, IRN; 3 Cardiology Medicine, Eisenhower Medical Center, Rancho Mirage, USA

**Keywords:** scad, multivessel scad, fmd and scad, scad types, scad management

## Abstract

Spontaneous coronary artery dissection (SCAD) is a relatively uncommon cause of acute coronary syndrome, which is mainly reported in postpartum patients and patients without typical cardiac risk factors. Our case was a 58-year-old female with a history of diabetes, hypertension, and hyperlipidemia who presented with non-exertional crushing retrosternal chest pain and was found to have ST elevation in inferior leads. Immediate cardiac catheterization was suggestive of spontaneous dissection of the third obtuse marginal artery, which was managed conservatively. Clinical suspicion is crucial for SCAD diagnosis, as it might be difficult to distinguish between coronary artery occlusion and SCAD. Moreover, revascularization in SCAD can be associated with complications. Therefore, SCAD needs to be considered as a differential diagnosis not only in patients without cardiac risk factors but also in patients with known cardiac risk factors like our case.

## Introduction

Spontaneous coronary artery dissection (SCAD) is an underdiagnosed cause of acute coronary syndrome, often requiring a high level of clinical suspicion for an accurate diagnosis [1]. Timely recognition is important, as invasive treatments can cause adverse outcomes. We present an intriguing case of SCAD with typical cardiac risk factors.

## Case presentation

A 58-year-old female with a history of hypertension, diabetes, and hyperlipidemia presented to the emergency department (ED) with a six-day history of cough, headache, dizziness, occasional shortness of breath, and non-exertional chest tightness. The physical exam was unremarkable. Her lab, including high-sensitivity troponin, was within normal limits. An electrocardiogram (EKG) revealed sinus rhythm with occasional premature ventricular contractions. The patient was discharged home.

**Figure 1:**
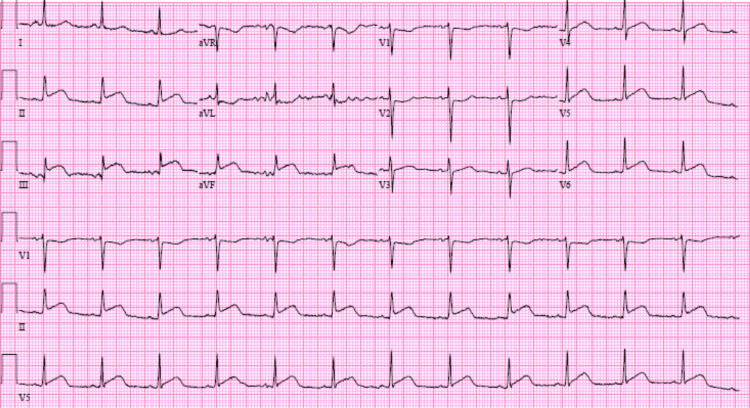
Inferior myocardial infarction. ST elevation in inferior leads (II, III, and AVF) with reciprocal ST depression in anterior leads (I and AVL). AVF: augmented vector foot, AVL: augmented vector left.

Two days later, she returned to the ED with non-exertional crushing retrosternal chest pain lasting 40 minutes, accompanied by shortness of breath and diaphoresis. Her blood pressure was 201/103 mmHg, respiratory rate was 14/min with 99% oxygen saturation on room air, temperature was 36.5°C, and heart rate was 78/min. The physical exam was unremarkable. Laboratory results showed mild leukocytosis. High-sensitivity troponin increased from 72 pg/ml to 9208 pg/ml, along with a B-type natriuretic peptide (BNP) of 196 pg/mL (Table 1). The EKG was consistent with an inferior infarction (Figure 1).

**Table 1:** Lab result at the time of admission and four hours later. BNP: B-type natriuretic peptide.

	Reference and unit	Initial result	Result after four hours
BNP	1-100 pg/ml	196 pg/ml	
High-sensitivity troponin	<14 pg/ml	72 pg/ml	9280 pg/ml

**Figure 2:**
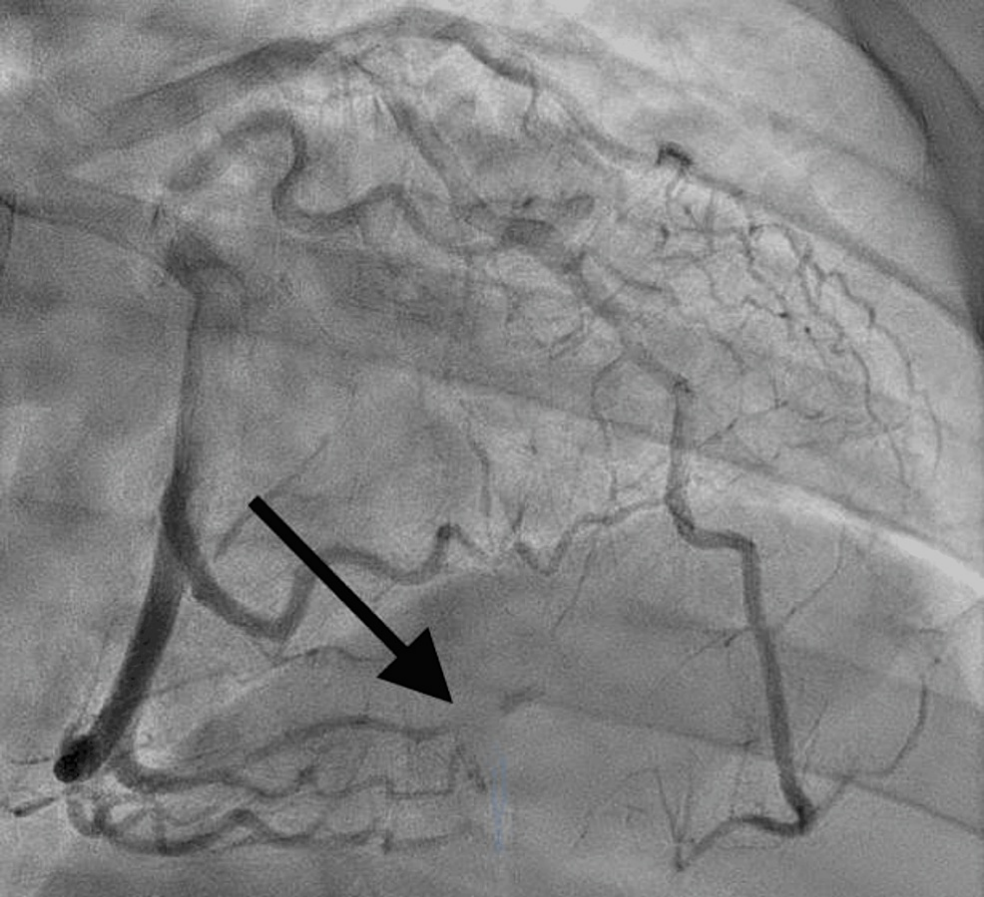
Third obtuse marginal SCAD (type 2b). SCAD: spontaneous coronary artery disease. Diffuse narrowing to the distal tip of the vessel (type 2b).

Immediate angiography revealed total occlusion of the third obtuse marginal artery in its midportion, with diffuse narrowing after the bifurcation and staining distally. The appearance was consistent with spontaneous coronary artery dissection type 2B (Figure 2). No percutaneous coronary intervention (PCI) was performed as she was stable and dissection happened in a small distal vessel. Transthoracic echocardiography (TTE) showed an ejection fraction of 60% with grade one diastolic dysfunction and no significant wall motion abnormalities. Blood pressure was managed with esmolol and nicardipine drips, and the patient was discharged on aspirin, spironolactone, carvedilol, and statin.

## Discussion

SCAD is characterized by non-traumatic, non-iatrogenic coronary artery dissection, leading to the formation of a false lumen that affects blood flow. Although its precise incidence is unknown, SCAD accounts for up to four percent of acute coronary syndrome cases, 43% of pregnancy-related myocardial infarctions, and 35% of acute coronary syndrome cases in women under 62 years of age [1]. It has been associated with female gender, pregnancy, infertility treatment, migraine headaches, hypothyroidism, and connective tissue diseases [1,2]. Fibromuscular dysplasia is present in 50% of SCAD patients [3]. Pregnancy-associated SCAD typically involves the left main coronary artery or the left anterior descending artery (LADA). However, SCAD in non-pregnant patients often affects multiple coronary arteries [4]. The stroke rate is higher in patients with multi-vessel SCAD compared to single-vessel SCAD [5]. 

Chest pain is the most common presenting symptom, with most patients initially presenting with ST-elevation myocardial infarction (STEMI) or non-ST-elevation myocardial infarction (NSTEMI). Less common manifestations include ventricular arrhythmias (5%), sudden cardiac death (0.8%), and cardiogenic shock (2%) [6]. STEMI presentation is associated with more severe stenosis and longer lesions, but no significant association exists between the initial presentation and immediate or late cerebrovascular and cardiac complications [7]. 

**Figure 3:**
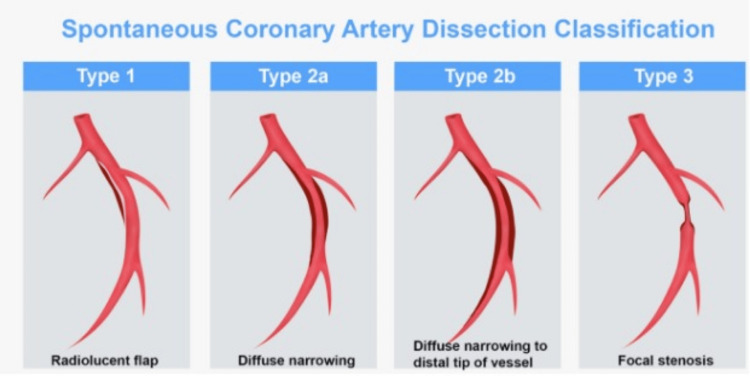
Spontaneous coronary artery dissection classification. Source: [8]. This article is licensed under a Creative Commons Attribution 4.0 International License, which permits use, sharing, adaptation, distribution, and reproduction in any medium or format, as long as you give appropriate credit to the original author(s) and the source.

Angiography is the gold standard method for diagnosis. It classifies SCAD into three types based on angiographic appearance. Type one is characterized by multiple lumens separated by a flap. Type two presents a long section of smooth stenosis along the vessel lumen, further divided into type 2a, where the caliber continues beyond the stenosis, and type 2b, where the stenosis extends to the most distal angiographic area. Type three is defined as a focal stenosis resembling atherosclerotic plaque (Figure 3) [8]. In types two and three, intracoronary artery imaging methods such as optical coherence tomography and intravascular ultrasound (IVUS) may be necessary [6]. However, in our case, no IVUS was performed due to the small size of the vessel. 

Currently, there are no established guidelines for SCAD treatment, with decisions made on a case-by-case basis. Conservative management, including beta-blockers, single antiplatelet therapy, and guideline-directed medical therapy (GDMT) in cases of decreased ejection fraction, is often preferred, as the vessel wall typically heals spontaneously. PCI is recommended for refractory ischemia, artery occlusion, and involvement of the main left coronary artery (LCA). Given the challenges associated with PCI, other interventions like balloon angioplasty, the "sandwich" technique, and cutting balloon angioplasty have been proposed [1,9]. Cutting balloon angioplasty, which creates fenestrations to decompress hematomas, has shown promise in restoring Thrombolysis in Myocardial Infarction Score (TIMI) three flow and improving clinical symptoms in a few studies [9,10].

In general, the prognosis of SCAD is good and the recurrence rate is approximately 10.4%, with blood pressure as the most common predictor, and beta-blockers offering a protective effect [1].

## Conclusions

SCAD is more prevalent than previously believed and should be considered in the differential diagnosis of acute coronary syndrome even in patients with typical cardiac risk factors. Conservative management is preferred in most stable cases. However, treatment strategies should be individualized.
